# Extracellular Total Electrolyte Concentration Imaging for Electrical Brain Stimulation (EBS)

**DOI:** 10.1038/s41598-017-18515-3

**Published:** 2018-01-10

**Authors:** Saurav Z. K. Sajib, Mun Bae Lee, Hyung Joong Kim, Eung Je Woo, Oh In Kwon

**Affiliations:** 1 Department of Biomedical Engineering, Kyung Hee University, Seoul, 02447 Korea; 2 Department of Mathematics, Konkuk University, Seoul, 05029 Korea

## Abstract

Techniques for electrical brain stimulation (EBS), in which weak electrical stimulation is applied to the brain, have been extensively studied in various therapeutic brain functional applications. The extracellular fluid in the brain is a complex electrolyte that is composed of different types of ions, such as sodium (Na^+^), potassium (K^+^), and calcium (Ca^+^). Abnormal levels of electrolytes can cause a variety of pathological disorders. In this paper, we present a novel technique to visualize the total electrolyte concentration in the extracellular compartment of biological tissues. The electrical conductivity of biological tissues can be expressed as a product of the concentration and the mobility of the ions. Magnetic resonance electrical impedance tomography (MREIT) investigates the electrical properties in a region of interest (ROI) at low frequencies (below 1 kHz) by injecting currents into the brain region. Combining with diffusion tensor MRI (DT-MRI), we analyze the relation between the concentration of ions and the electrical properties extracted from the magnetic flux density measurements using the MREIT technique. By measuring the magnetic flux density induced by EBS, we propose a fast non-iterative technique to visualize the total extracellular electrolyte concentration (EEC), which is a fundamental component of the conductivity. The proposed technique directly recovers the total EEC distribution associated with the water transport mobility tensor.

## Introduction

Electrical brain stimulation (EBS) techniques have emerged as a potential treatment for psychiatric disorders, including transcranial direct current stimulation (tDCS), cranial electrotherapy stimulation (CES), electroconvulsive therapy (ECT), and deep brain stimulation (DBS)^1,2^. The tDCS typically delivers a weak electrical current for approximately 20 minutes targeting brain areas, and has been reported to improve a wide range of neurological and psychiatric disorders^3,4^. CES is a non-invasive brain stimulation technique used for treating conditions such as anxiety, depression, and insomnia^5^. Currently, DBS is an effective and increasingly popular treatment in specific brain regions for a variety of movement disorders, including dystonia and pain. Unfortunately, due to limitations in imaging capabilities, the exact effect of electrical stimulation on brain tissue has many problems to be solved^6,7^.

The ionic concentration of the extracellular space (ECS) is altered by the energy status and cellular integrity, which is an effective indicator of the disease state^8,9^. The concentrations of sodium (Na^+^), potassium (K^+^) and calcium (Ca^2+^) ions are known homeostatic aspects of the human body. Many diseases are the result of homeostatic imbalance. The contrast of ion concentrations in different tissues is necessary to detect pathologies that distinguish normal and diseased tissues. The *in vivo* non-invasive measurement of ECS ion concentrations, which is directly related to the electrical conductivity, is quite challenging in patients with neurological diseases^10^.

The current injected into a region of interest (ROI) of the brain through a direct current stimulation produces an internal current density distribution. MRI scanners have been used since the 1990’s to investigate electrical properties, including conductivity, permittivity, and current density distributions inside the human body^11–13^. Magnetic resonance electrical impedance tomography (MREIT) is a method for visualizing the internal current density by measuring one component of the magnetic flux density using an MRI scanner^14–16^. The MREIT technique provides a tool for investigating the electrical properties of EBS in the brain.

Diffusion tensor imaging is an MR imaging modality using the Brownian motion of water within biological tissues, which has been used extensively to map the neural axons of white matter in the brain^17–20^. Diffusion-weighted MRI has become a popular method to directly obtain information about tissue microstructure and connectivity of the brain. To distinguish the water compartments of biological tissue, the apparent diffusion coefficient (ADC) technique measures a signal loss due to the diffusion of water molecules in the tissue. The diffusion of water molecules in the extracellular space depends on the geometry of the cellular elements, volume fraction, size of the molecules, viscosity of the medium, and orientation, etc. The non-invasive investigation of water diffusion quantitatively characterizes the fine structural features and geometric organization of the neural tissues.

The MREIT technique measures the magnetic flux density by externally injected current using an MRI scanner and visualizes the apparent current density distribution inside an imaging object. The current density distribution reflects the ECS electrical properties in the brain region, including conductive fluids of numerous ions and ion-exchangeable heterogeneous membrane structures. Electrical conductivity in the extracellular brain region is determined as a sum of products of the carrier concentrations and mobilities. The effective macroscopic anisotropic conductivity tensor approximately shares eigenvectors with the water diffusion tensor in terms of the intra- and extra-cellular transport coefficients by a two-phase anisotropic medium^21^. By adopting the linear relationship between the conductivity tensor and the water diffusion tensor^22,23^, the anisotropic conductivity tensor is recovered by combining the diffusion-tensor MRI and MREIT techniques without any referred extracellular information^24^.

To characterize the ion concentration in the ECS, we reasonably assume that the mobility of charge carriers is proportional to the mobility of water molecules in the same structural environment. The non-invasive investigation of water diffusion can be linked to the mobility of charge carriers through Einstein’s relationship, where the diffusivity of moving particles in a fluid is related to the mobility. The diffusion tensor MREIT (DT-MREIT) technique, which is based on the linear relationship between the water diffusion tensor and the electrical conductivity tensor, directly recovers the anisotropic conductivity tensor combined with the water mobility tensor induced by the diffusion tensor. As an application of DT-MREIT, it is possible to describe specifically the electrical properties during ECS in the brain area. The recovered apparent conductivity tensor indicates electrical properties that can be expected from DT-MREIT using the reconstructed current density. However, the internal conductivity can have an infinite number of combinations of ion concentration and mobility. The total extracellular electrolyte concentration (EEC) distribution is one component of the conductivity at a low frequency. We analyze the relation between the concentration of ions and the electrical properties (conductivity and current denstiy) extracted from the magnetic flux density measurements. By measuring the magnetic flux density induced by EBS and ADCs, we propose a fast non-iterative technique to visualize the total EEC. To reliably separate EEC from the conductivity, we determine an optimal regularization parameter for the weighted least square problem using the generalized cross-validation (GCV) method, which minimizes the predictive mean-square error without statistical information^25^. The proposed technique directly recovers the total EEC distribution with respect to the diffusion behavior of water molecules.

To demonstrate the proposed method, two types of isotropic phantom experiments were conducted for the proposed ion concentration imaging. A cylindrical acrylic cage filled with an agar in the background region was used in both phantom experiments. The first phantom experiment was designed to demonstrate the influence of the medium’s properties on the reconstructed EEC image by adding polyvinylpyrrolidone (PVP) material. The viscosity of PVP does not affect EEC without chemical bonding and only affects the mobility property of the anomalies. The second phantom was designed to demonstrate the effect of the ion size and mass on the reconstructed EEC image. To test the noise resistance of the proposed method, we artificially degraded the measured magnetic flux density for the two phantom experiments and compared the reconstructed ion concentration and conductivity images. Animal experiments with a healthy beagle were conducted. To visualize the EEC distribution, we recovered the current density inside the brain region using the measured magnetic flux density caused by EBS. We separated the total EEC distribution from the conductivity tensor map by combining the water ion mobility information. The animal experiment shows that the total EEC has the potential to visualize new electrical properties for EBS, including the current pathway, electric field distribution, apparent ion velocity in the electric field, and anisotropic conductivity tensor.

## Results

### Phantom experiments setup

**Figure 1:**
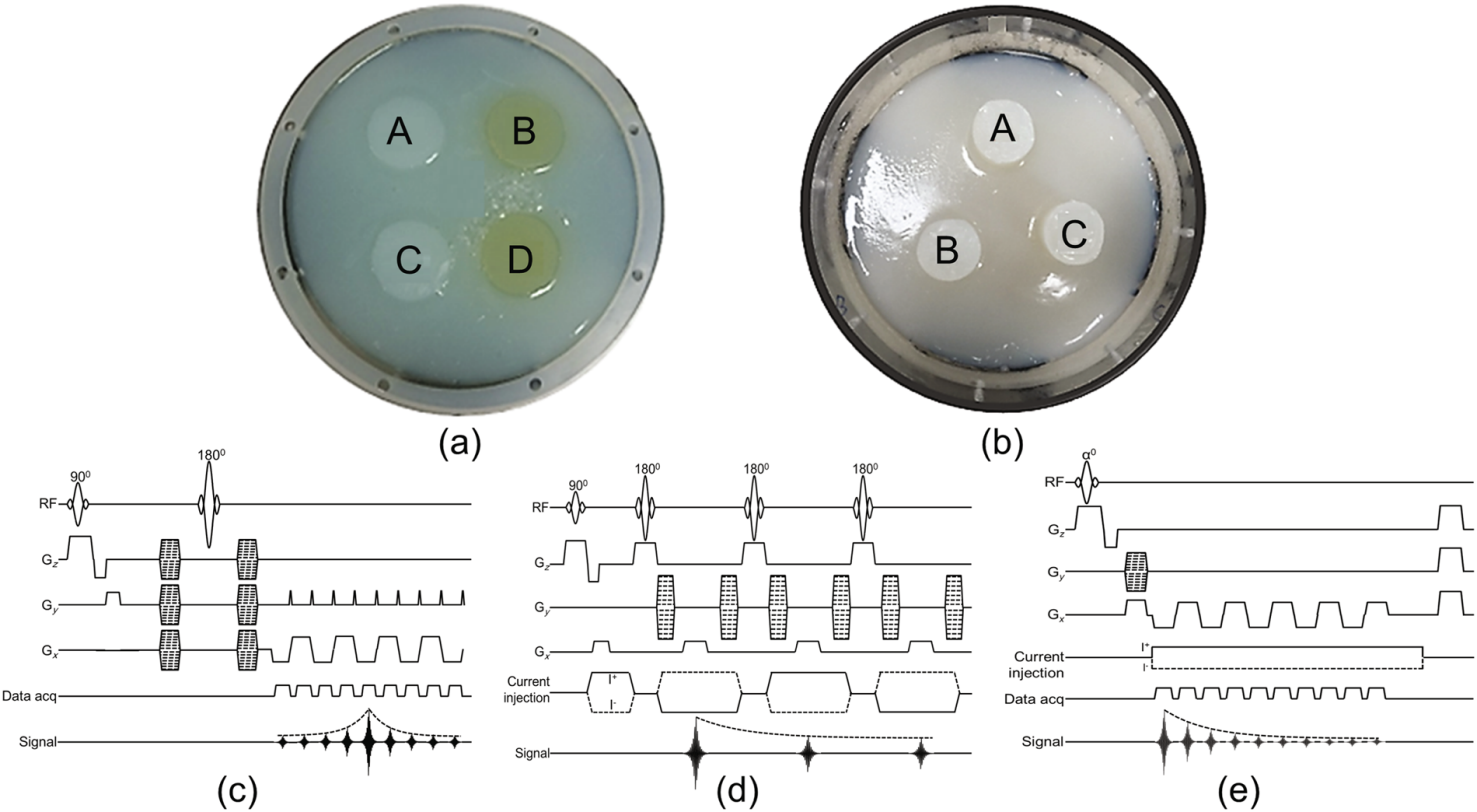
Experimental phantom photograph (**a**) with four anomalies (phantom-1) and (**b**) with three anomalies (phantom-2). (**c**) Single-shot spin-echo echo planar imaging pulse sequence for diffusion weighted imaging. (**d**) and (**e**) Multi-spin echo and multi-gradient echo MR pulse sequence synchronized with current injection to measure externally induced *B*_*z*_ data, respectively.

Two different experimental phantoms were constructed to demonstrate the proposed ion concentration imaging (Fig. 1). A cylindrical acrylic cage with a 16 cm diameter and 12 cm height filled with an agar in the background region was used in both phantom experiments. Phantom-1 (Fig. 1(a)) was designed to demonstrate the influence of the medium’s properties on the reconstructed ion concentration image. Four different anomaly regions were made by varying the NaCl concentration or medium viscosity characteristics. For anomalies A and B positioned at phantom-1 (Fig. 1(a)) 3.75 g/L NaCl was mixed with 25 g/L agar and 1.25 g/L CuSO_4_, whereas for anomalies C and D, 10 g/L NaCl was mixed with the same amount of agar and CuSO_4_ solution. To control the mobility of both the ions and water molecules, we added PVP, approximately 100 g/L, inside the anomalies located at B and D only.

Phantom-2 (Fig. 1(b)) was designed to demonstrate the effect of the ion size and mass on the reconstructed ion concentration image. For that purpose, we created three different anomalous regions. The anomaly positioned at A was constructed with 50 mM NaCl (2.9 g/L NaCl, 25 g/L agar, 1.25 g/L CuSO_4_), whereas, the anomaly located at B contained the same molarity of choline (7.0 g/L choline chloride, 25 g/L agar, 1.25 g/L CuSO_4_). The concentrations of ions were the same for both anomalies A and B. The anomaly at position C was a mixture of NaCl and choline in a 2:1 ratio. The conductivity was measured for all seven samples using a conductivity meter (Solatron, USA) at a frequency of 10 Hz. For the A, B, C, and D (Fig. 1(a)) anomalies of phantom-1, the conductivity value was 0.97, 0.69, 2.09, and 1.81S/m, respectively. For phantom-2 (Fig. 1(b)), the value of the conductivity was 0.74 (A), 0.63 (B), and 1.63 (C) S/m, respectively.

The imaging experiment was performed using a 3 T Phillips MR scanner (Acievea, Netherlands) equipped with an 8-channel head coil installed in Kyung Hee University Hospital. Using a custom designed current source^26^, we injected 10 mA current sequentially through a pair of horizontal and vertical electrodes (Fig. 1(a) and (b)) for both phantoms. We acquired the z-th magnetic flux density data, ${B}_{z}^{i}$ (*i* = 1, 2), on five imaging slices using a multi-spin echo MR pulse sequence (Fig. 1(d)) with a spatial resolution of 1.875 × 1.875 × 10 mm^3^. The other imaging parameters were as follows: TR/TE, 1000/18 ms, number of echoes (*N*_*E*_ = 3), number of slices (*N*_*s*_ = 5), number of averages (NEX = 20), and field of view (FOV = 240 × 240 × 50 mm^3^). The reconstructed images were displayed on the third imaging slice. We also measured the diffusion of water molecules by using the single-shot spin-echo echo planar imaging (SS-SE-EPI) pulse sequence (Fig. 1(c)) with a *b*-value of 1000 s/mm^2^ and fifteen gradient directions with a spatial resolution of 3.75 × 3.75 × 10 mm^3^. The imaging parameters of the diffusion experiment were as follows: TR/TE = 2000/71 ms, FOV = 240 × 240 × 50 mm^3^, and NEX = 1. The collected diffusion data sets were interpolated to the spatial resolution of the MREIT experiment.

**Figure 2:**
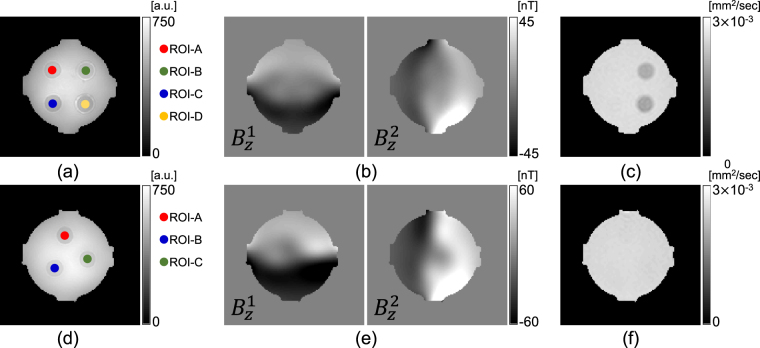
(**a**) and (**d**) Echo combined MR magnitude images acquired during the MREIT experiment for phantom-1 and 2, respectively. The ROI marked in circles was used to measure the reconstructed total electrolyte concentration and the corresponding diagonal component of the conductivity tensor values in Table 1. (**b**) and (**e**) ${B}_{z}^{i}$ (*i* = 1, 2) images induced by an external current injection of 10 mA for the phantom, respectively. (**c**) and (**f**) ADC map obtained from the diffusion experiment at a *b*-value of 1000 s/mm^2^.

Figure 2(a,b,d,b and e) show the acquired MR magnitude and *B*_*z*_ images acquired for both directions from the center slices of phantom-1 and 2, respectively. The ADC obtained from the DWI experiment is displayed in Fig. 2(c) and (f). As seen from the reconstructed ADC map (Fig. 2(c)), the mobility of water molecules is influenced by the medium viscosity property of the aqueous PVP solution.

### Animal experiments setup

We also demonstrated the total electrolyte ionic concentration imaging using *in vivo* animal subject data from the Impedance Imaging Research Center (http://iirc.khu.ac.kr/software) which is available for non-commercial use. For convenience, we briefly describe the experimental setup. We injected 0.1 mg/kg of atropine sulfate to anesthetize the dog with an intramuscular injection of 0.2 ml/kg Zolazepam (Zoletil 50, Virbac, France). Two pairs of carbon-hydrogel (HUREV Co. Ltd, Korea) surface electrodes were attached to the skin. All of the experimental protocols were approved by the institutional animal care and use committee of Kyung Hee University (KHUASP-14-25). A 2 mA current was injected horizontally and vertically using a custom designed MREIT current source. We acquired MR images using a coherent steady state multi-gradient echo (CSS-MGRE) pulse sequence (Fig. 1(e)) with a spatial resolution of 1.25 × 1.25 × 5 mm^3^. The imaging parameters were as follows: repetition time TR = 200 ms, echo time TE = 2.3 ms, number of echoes (*N*_*E*_ = 13), number of slices (*N*_*s*_ = 6), flip angle 40°, FOV = 160 × 160 mm^2^, NEX = 35, and imaging matrix size 128 × 128. We performed DT-MRI scans using the single-shot spin-echo echo planar imaging (SS-SE-EPI) pulse sequence to measure the diffusion tensor map. We applied the diffusion-weighting gradients in 32 directions with a *b*-value of 800 s/mm^2^. The imaging parameters were TR/TE = 8000/94 ms, slice thickness = 5 mm, NEX = 2, and FOV = 160 × 160 mm^2^, and the acquisition matrix size was 112 × 112. The total scan time to collect one set of diffusion weighted images was approximately 8.8 min.

The *j*-th MR signal, *S* ^*j*^, was combined as a complex sum of MR signals of multi-channel receive coils. For the measured magnetic flux density data, *B*_*z*_,_*j*_,*j* = 1, …, *N*_*E*_, a magnetic flux density was generated by a weighted combination, ${B}_{z}={\sum }_{j\mathrm{=1}}^{{N}_{E}}{w}_{j}{B}_{z,j}$. The weighting factor *w*_*j*_ was

1 $${w}_{j}=\frac{{|{T}_{{c}_{j}}{S}^{j}|}^{2}}{\sum _{k=1}^{{N}_{E}}{|{T}_{{c}_{k}}{S}^{k}|}^{2}},\,j\,=\,\mathrm{1,}\cdots ,{N}_{E}$$

where ${T}_{{c}_{j}}$ denotes the time width of injection current for the *j*-th echo^27^.

### Results of phantom experiments

**Figure 3:**
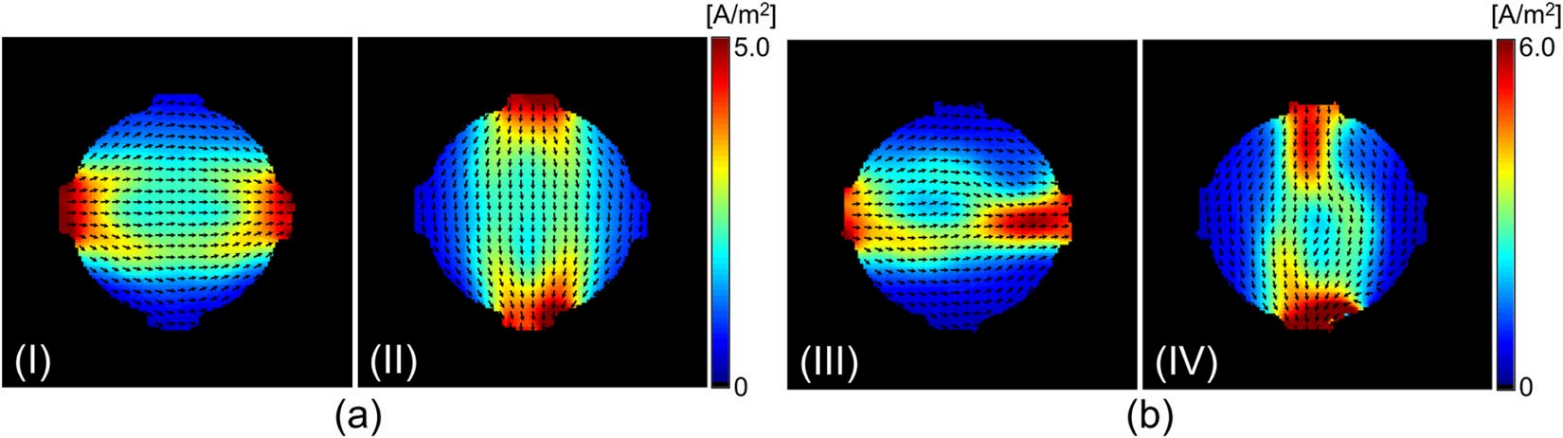
(**a**) and (**b**) Intensity of the projected current density in both directions (I and II), recovered from the measured magnetic flux density in Fig. 2(b) and (e), respectively. The normalized arrow plots show the direction of the current flow.

**Figure 4:**
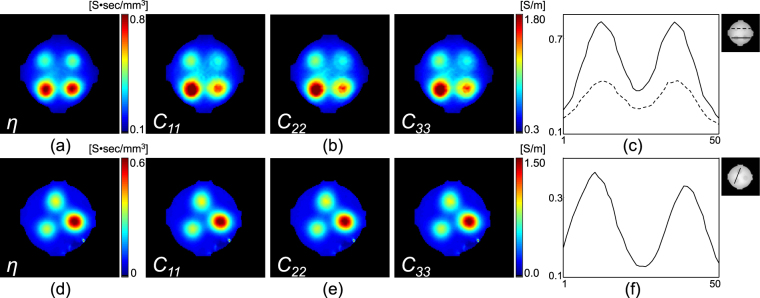
(**a**) and (**d**) Reconstructed ion concentration images for phantom-1 and 2, respectively. (**b**) and (**e**) Corresponding diagonal components of the conductivity tensor. (**c**) and (**f**) Profile plots of the reconstructed ion concentration images.

With the measured magnetic flux density data, ${B}_{z}^{i}\mathrm{,\ }i=\mathrm{1,}\,2$ (Fig. 2(b) and (e)), we first recovered the projected current density, ${{\bf{J}}}^{P,i}={{\bf{J}}}^{\mathrm{0,}i}+(\frac{\partial {\psi }^{i}}{\partial y},-\frac{\partial {\psi }^{i}}{\partial x},\,0)$, by solving equation (14). Figure 3(a) and (b) show the estimated projected current density $(\Vert {{\bf{J}}}^{P,i}\Vert \mathrm{,\ }i=\mathrm{1,}\,2)$ for both phantoms. Using the current density estimated from the measured magnetic flux density, combined with the water diffusion tensor data in equation (16), we reconstructed the total ion concentration distribution on the center imaging slice for the both phantoms, as shown in Fig. 4(a) and (d). Since the structure of the generated phantoms is simple, we directly recovered the total ion concentration by solving the matrix system (17). By multiplying the total ion concentrations with the water diffusion tensor, we reconstructed the conductivity tensor. Figure 4(b) and (e) show the reconstructed diagonal components of the conductivity tensor.

The recovered conductivity values in the C and D (A and B) regions of phantom-1 were 1.74 and 1.34 S/m (0.98 and 0.81 S/m), respectively, which indicates the electrical property that can be expected from the MREIT technique with reconstructed current density information. However, there can be an infinite number of combinations of ion concentration and mobility that result in the same conductivity value. The electrical conductivity is proportional to the product of the mobility and the carrier concentration. We separated the anisotropic conductivity tensor into the EEC and water mobility terms in (13). By this decomposition, the same conductivity can occur from different numbers of electrons having different mobilities. We provide the total EEC and mobility, which are two components of conductivity. For phantom-1, the electrical ionic concentrations were the same in anomalies A and B (C and D) (Table 1). Therefore, the reconstructed total concentration images (Fig. 4(a) and (c)) show similar contrast at the ROIs A and B (C and D) Table 2. Because the mobility for the anomalies were located at B and D being controlled by the PVP, the reconstructed conductivity exhibits a different conductivity contrast at the four different anomalous locations. The ratio between the reconstructed concentration values in the ROIs A and B (C and D) (Fig. 4(a)) was 1.75 (0.42/0.24), which was similar to the ratio of 2.7 from the samples (Table 1).

**Table 1:** Summary of the material used for preparing the phantom experiment. The conductivity was measured before the experiment using the four electrode method (Solatron, USA) at a frequency of 10 Hz.

	Phantom-1				Phantom-2		
	ROI-A	ROI-B	ROI-C	ROI-D	ROI-A	ROI-B	ROI-C
NaCl [g/L]	3.75	3.75	10.0	10.0	2.90	0	5.80
Agar [g/L]	25.0	25.0	25.0	25.0	25.0	25.0	25.0
CuSO_4_ [g/L]	1.25	1.25	1.25	1.25	1.25	1.25	1.25
PVP [g/L]	0	100	0	100	0	0	0
Choline chloride [g/L]	0	0	0	0	0	7	7
Conductivity [S/m]	0.97	0.69	2.09	1.81	0.74	0.63	1.63

**Table 2:** Parameters for imaging experiments.

Experiment	Pulse sequence	Resolution (mm^3^)	FOV (mm^3^)	TR/TE (ms)	*N*_*E*_/NEX
MREIT (phantom)	MSE with 10 mA current	1.875 × 1.875 × 10	240 × 240 × 50	1000/18	3/20
MREIT (canine)	CSS-MGRE with 2 mA current	1.250 × 1.250 × 5	160 × 160 × 30	300/2.3	13/35
DWI (phantom)	SS-SE-EPI (*b* = 1000 s/mm^2^ ×, 15 directions)	3.750 × 3.750 × 10	240 × 240 × 50	2000/71	1/1
DWI (canine)	SS-SE-EPI (*b* = 800 s/mm^2^, 32 directions)	1.430 × 1.430 × 5	160 × 160 × 30	8000/94	1/2

However, the reconstructed ion concentration images in phantom-2 show some differences. Choline is 9.7% lower than NaCl for the anomaly located in A and B, even though we mixed the same amount of NaCl and choline. We speculate that this difference is caused by the dependency of the activity coefficient, which appeared in equation (13). The activity coefficient of individual ionic species is different because it is related to the dissociation factor in an electrolyte solution, and its value is between 0 and 1^28^. The measured macroscopic conductivity value also shows a similar tendency (Fig. 1(b)). Numerical values of the reconstructed ion concentration and diagonal components of the conductivity tensor measured within the ROIs marked in Fig. 2(a) and (d) are summarized in Table 3.

**Table 3:** Numerical values of the reconstructed EEC image and diagonal components of the conductivity tensor measured within the ROIs marked in Fig. 2(a) and (d).

	Phantom-1				Phantom-2		
	ROI-A	ROI-B	ROI-C	ROI-D	ROI-A	ROI-B	ROI-C
*η* [S · sec/mm^3^]	0.39 ± 0.03	0.39 ± 0.04	0.68 ± 0.09	0.68 ± 0.07	0.31 ± 0.03	0.29 ± 0.03	0.52 ± 0.07
C_11_ [S/m]	0.99 ± 0.08	0.82 ± 0.07	1.76 ± 0.21	1.36 ± 0.11	0.79 ± 0.09	0.73 ± 0.07	1.31 ± 0.17
C_22_ [S/m]	0.97 ± 0.07	0.81 ± 0.07	1.72 ± 0.21	1.34 ± 0.12	0.79 ± 0.08	0.73 ± 0.07	1.29 ± 0.16
C_33_ [S/m]	0.98 ± 0.08	0.81 ± 0.07	1.74 ± 0.22	1.33 ± 0.12	0.78 ± 0.08	0.73 ± 0.08	1.31 ± 0.16

**Figure 5:**
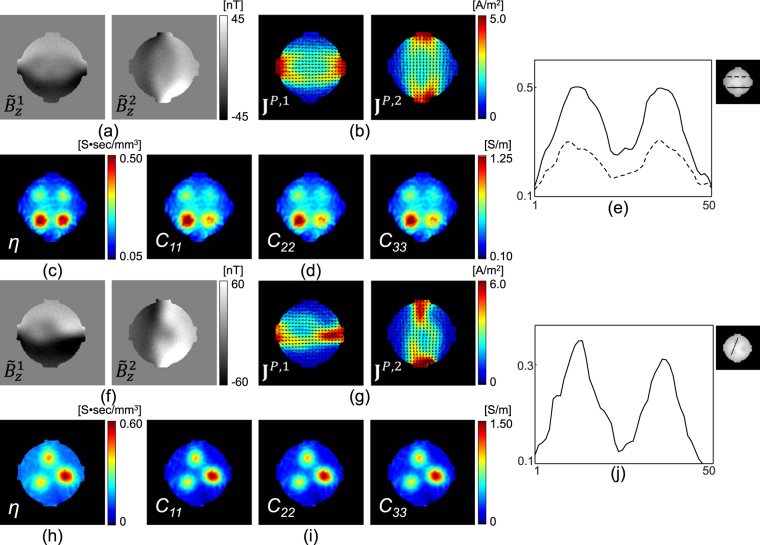
Phantom experiment results by adding random noise. (**a**) and (**f**) Degraded ${\tilde{B}}_{z}^{i}\mathrm{,\ }i\,=\,\mathrm{1,2,}$ images for phantom-1 and 2, respectively. (**b**) and (**g**) Denoised projected current density images from the noisy ${\tilde{B}}_{z}^{i}\mathrm{,\ }i\,=\,\mathrm{1,2,}$ data. (**c**) and (**h**) Reconstructed ion concentration images for phantom-1 and 2, respectively. (**d**) and (**i**) Corresponding diagonal components of the conductivity tensor. (**e**) and (**j**) Profile plots of the reconstructed ion concentration images.

To evaluate the effect of noise on the ion concentration and conductivity images, we added artificial noise to the measured magnetic flux density data, ${B}_{z}^{i}\mathrm{,\ }i=1,\,2$. We evaluated the noise level of the measured ${B}_{z}^{i}$ for the both phantoms. The noise level, $s{d}_{{B}_{z}}$, was evaluated from the homogeneous background region as the following^29^:

2 $$s{d}_{{B}_{z}}=\frac{s{d}_{{\nabla }^{2}{B}_{z}}}{\sqrt{\frac{1}{{{\rm{\Delta }}}^{4}}+\frac{1}{{{\rm{\Delta }}}_{z}^{4}}}}$$

where Δ, Δ_*z*_, and $s{d}_{{\nabla }^{2}{B}_{z}}$ are the pixel size, slice thickness, and standard deviation of Laplacian of *B*_*z*_, respectively. The standard deviations of the measured noise included in the measured magnetic flux density for phantom-1 and phantom-2 were 0.39 and 0.88 nT, respectively. We artificially degraded the measured ${B}_{z}^{i}$ data by adding uniform random noise

3 $${\tilde{B}}_{z}^{i}={B}_{z}^{i}+2\times 9\times s{d}_{{B}_{z}}\times ({r}_{n}-\frac{1}{2})$$

where *r*_*n*_ denotes a random number uniformly distributed in the range from 0 to 1. We set the measured ${B}_{z}^{i}={B}_{z}^{i,true}+2\times s{d}_{{B}_{z}}\times ({r}_{n}-\frac{1}{2})$ for the noiseless ${B}_{z}^{i,true}$ because the measured ${B}_{z}^{i}$ already contains noise artifacts with the noise level of $s{d}_{{B}_{z}}$. The generated magnetic flux density ${\tilde{B}}_{z}^{i}\mathrm{,\ }i=1,2$, contain noise artifacts corresponding to 1 mA current injection. Figure 5 shows the reconstructed results corresponding to Fig. 4. Figure 5(a) and (f) show the degraded ${\tilde{B}}_{z}^{i}\mathrm{,\ }i=1,\,\mathrm{2,}$ images for phantom-1 and 2, respectively. To suppress the noise effects on the reconstructed projected current densities J^*P,I*^, *i* = 1, 2, we used the anisotropic diffusion-based denoising approach derived from the Perona-Malik nonlinear diffusion model^30^. Figure 5(b) and (g) show the denoised projected current density estimated from the noisy ${\tilde{B}}_{z}^{i},\,i=1,\,2$. The reconstructed EEC map and its corresponding diagonal components of the conductivity tensor are displayed in Fig. 5(c,d,h,i)) for phantom-1 (2), respectively. Figure 5(e) and (j) show the profile plots of the reconstructed ion concentration images.

### Results of animal experiments

**Figure 6:**
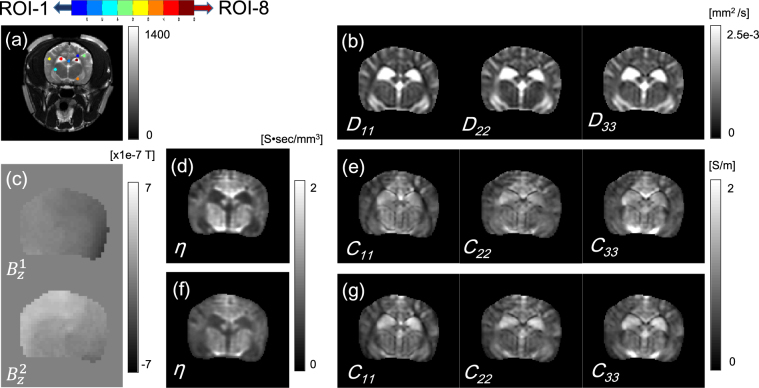
(**a**) MR magnitude image with four positions of the current injection electrodes. (**b**) Diagonal components of the diffusion tensor. (**c**) Magnetic flux densities by injected currents. (**d**) and (**e**) Computed EEC and diagonal components of the reconstructed conductivity tensors with *λ*_*cond*_, respectively. (**f**) and (**g**) Computed EEC and diagonal components of the reconstructed conductivity tensors with *λ*_*opt*_, respectively.

Figure 6(a) shows the MR magnitude image of the brain region from the MREIT scan with four positions of the current injection electrodes. Figure 6(b) shows the diagonal components of the diffusion tensor at the second slice position. We recovered the projected current density inside the brain region from the measured magnetic flux density (Fig. 6(c)). Using the estimated current density, we reconstructed the total EEC on the center imaging slice by combining the water diffusion tensor.

We chose eight ROIs, while considering the tissue anisotropy characteristics (white matter (ROI-1, 2, 3), gray matter (ROI-4, 5, 6), and CSF (ROI-7, 8)). To evaluate the performance of the proposed method, we chose a search neighborhood ${{\mathscr{N}}}_{(x,y)}=\{({x}_{i},{y}_{i}):i=1,\cdots ,\mathrm{9\}}$ around an imaging pixel (*x*, *y*). Although the matrix formula for EEC in (18) is clear, due to the relatively noisy diffusion tensor data and the inversion process using *D* ^−1^, the inversion process of matrix *A* is severely unstable. The regularization parameter *λ* is critical to determining the EEC by solving the over-determined system *A*_*x*_ = *b* ^31^. We chose *λ*_*cond*_ as the average of the condition numbers of the matrix *A* in (18). By multiplying EEC (obtained using *λ*_*cond*_) by the water diffusion tensor, we reconstructed the conductivity tensors. The values of the diagonal components of the reconstructed anisotropic conductivity tensors in those eight ROIs are summarized in Table 4. The resulting EEC image and the diagonal components of the conductivity tensors for the regularization parameter *λ*_*cond*_ are shown in Fig. 6(d) and (e), respectively. In this case, the reconstructed conductivity values were relatively small in the CSF regions (ROI-7, 8). For example, the ratio between the conductivity values in ROI-8 and ROI-6 was 1.8 (1.08/0.60). Moreover, the conductivity value in ROI-8 was 1.08 S/m, which was smaller than the literature value of approximately 1.8S/m, which was measured from extracted samples by using the four-electrode method at 37 °C^32^.

**Table 4:** Estimated values of the reconstructed ion concentration and the diagonal components of the conductivity tensor measured within the ROIs using the regularization parameters *λ*_*cond*_ and *λ*_*opt*_, respectively.

		ROI-1	ROI-2	ROI-3	ROI-4	ROI-5	ROI-6	ROI-7	ROI-8
*λ*_*cond*_	*η* [S · sec/mm^3^]	0.80 ± 0.14	0.90 ± 0.15	0.76 ± 0.07	0.56 ± 0.06	0.32 ± 0.04	0.61 ± 0.05	0.43 ± 0.01	0.36 ± 0.02
	C_11_ [S/m]	0.27 ± 0.07	1.43 ± 0.49	0.46 ± 0.04	0.54 ± 0.04	0.44 ± 0.06	0.59 ± 0.07	1.18 ± 0.02	1.07 ± 0.05
	C_22_ [S/m]	0.93 ± 0.14	0.48 ± 0.14	0.85 ± 0.03	0.55 ± 0.06	0.44 ± 0.06	0.60 ± 0.07	1.30 ± 0.06	1.10 ± 0.06
	C_33_ [S/m]	0.41 ± 0.11	0.74 ± 0.08	0.55 ± 0.04	0.57 ± 0.05	0.45 ± 0.05	0.61 ± 0.08	1.29 ± 0.06	1.07 ± 0.06
*λ*_*opt*_	*η* [S · sec/mm^3^]	0.84 ± 0.10	0.86 ± 0.08	0.65 ± 0.03	0.62 ± 0.05	0.46 ± 0.04	0.61 ± 0.03	0.50 ± 0.01	0.54 ± 0.02
	C_11_ [S/m]	0.29 ± 0.09	1.36 ± 0.37	0.40 ± 0.03	0.60 ± 0.07	0.63 ± 0.06	0.59 ± 0.09	1.39 ± 0.03	1.62 ± 0.06
	C_22_ [S/m]	0.97 ± 0.12	0.46 ± 0.10	0.73 ± 0.05	0.61 ± 0.09	0.63 ± 0.07	0.60 ± 0.09	1.53 ± 0.06	1.67 ± 0.06
	C_33_ [S/m]	0.44 ± 0.13	0.72 ± 0.09	0.48 ± 0.04	0.63 ± 0.08	0.64 ± 0.05	0.62 ± 0.09	1.52 ± 0.06	1.63 ± 0.08

We minimized the GCV function *GCV*(*λ*) in (19) to determine an optimal regularization parameter at each voxel. Figure 6(f) shows the reconstructed EEC image in the brain region, which was different from the MR magnitude image in Fig. 6(a). The low-frequency electrical conductivity tensor images by the proposed method with *λ*_*opt*_ are given in Fig. 6(g), which corresponds to the diffusion tensor in Fig. 6(b). The values of the reconstructed EEC optimized by the regularization parameter *λ*_*opt*_ and the diagonal components of the conductivity tensor measured within the ROIs are displayed in Table 4. The EEC values and conductivity values in the CSF regions were different depending on the regularization parameters *λ*_*cond*_ and *λ*_*opt*_, respectively. Using *λ*_*opt*_, the ratio between the conductivity values in ROI-8 and ROI-6 was 2.7 (1.64/0.60) and the conductivity value in CSF was reasonably close to the expected value.

**Figure 7:**
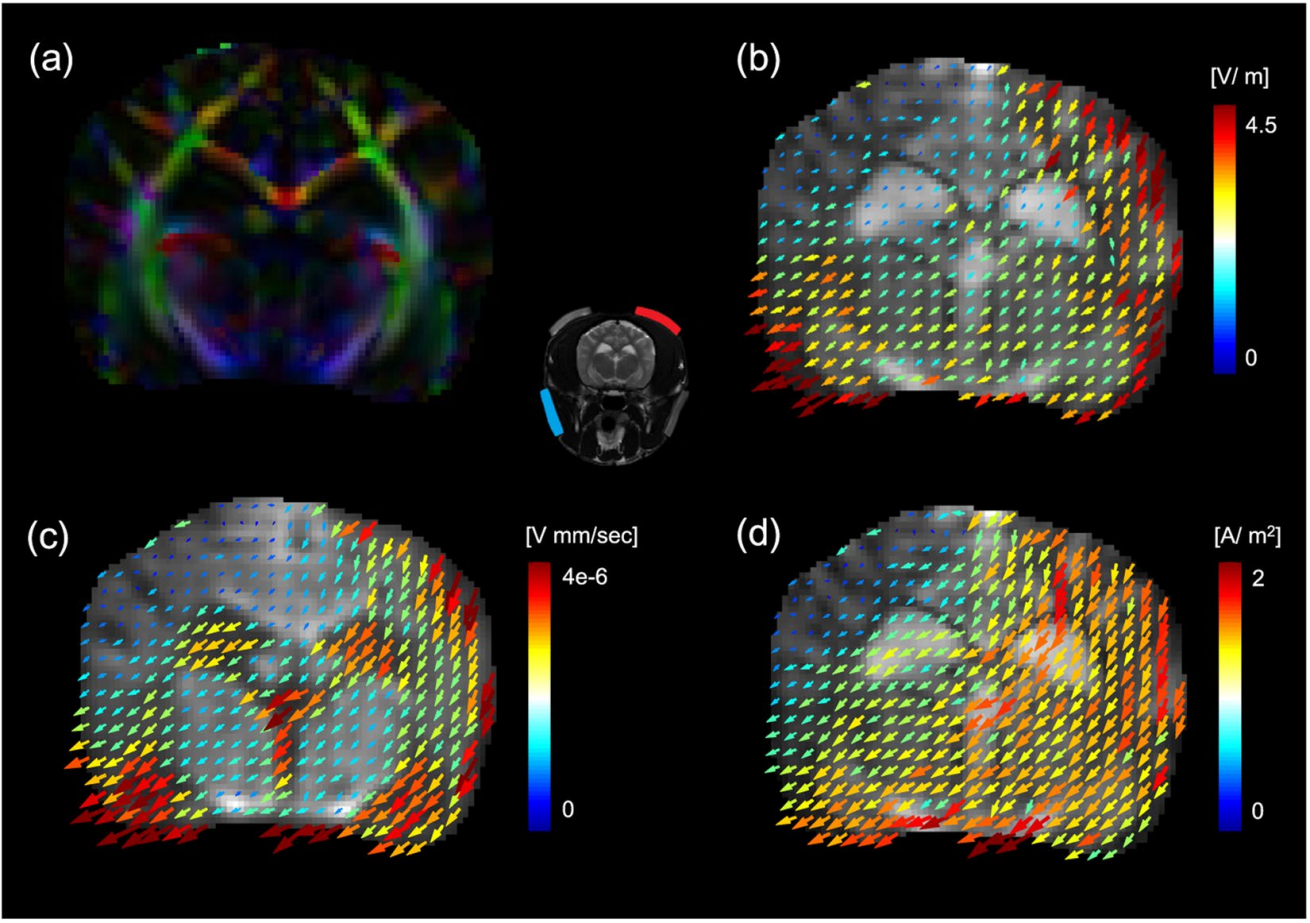
(**a**) Color-coded fractional anisotropic (FA) map. (**c**) Image of EEC overlaid on arrows that indicate the directions and magnitudes of the velocity vectors. (**b**) and (**d**) Image of the mean of the diagonal components of the reconstructed conductivity tensor on arrows that indicate the electric field and the recovered current density, respectively. The central image shows the electrode montage used for the corresponding current injection.

We note that the EEC values of the CSF regions were relatively lower than those of the other regions despite the high conductivity values of the CSF regions. The EEC value in the CSF region and that in the other regions was 0.52 S. sec/mm^3^ and 0.67 S. sec/mm^3^, respectively. Figure 7 shows that the conductivity value in the CSF region was higher than that in other brain regions because the concentration of ions was relatively low but the velocity of ions was relatively fast.

Using the acquired DT-MRI scan data, we visualized the color-coded fractional anisotropy (FA) map in the brain regions in Fig. 7(a). We calculated the mean of the diagonal components of the reconstructed conductivity tensor; $\hat{c}=\frac{{c}_{11}+{c}_{22}+{c}_{33}}{3}$. Figure 7(b) shows the directions and magnitudes of the recovered electric field $E=-\nabla u$ that corresponds to EBS overlaid on the image of $\hat{c}=\frac{{c}_{11}+{c}_{22}+{c}_{33}}{3}$. The electrode montage is displayed in the center position in Fig. 7. The image of $\hat{c}$ in Fig. 7(d) was overlaid on the arrows that indicate the direction and magnitudes of the recovered current density. Figure 7(d) shows the relationship between the EEC, anisotropic conductivity tensor, and current density. Since the EEC information is a primary component of the conductivity, the recovered EEC can provide the electric velocity of the ions in the ECS caused by the applied EBS. Figure 7(c) shows the velocity of the total ions in the ECS for the applied EBS.

**Figure 8:**
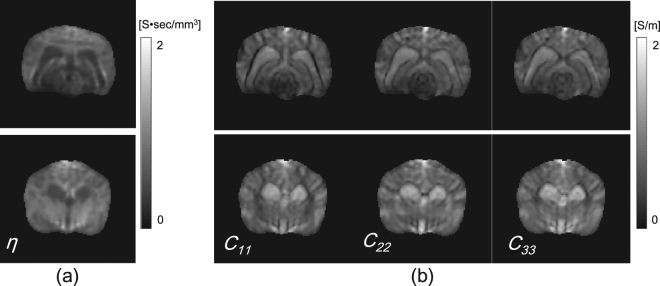
(**a**) and (**b**) Recovered EEC and diagonal components of the reconstructed conductivity tensors images using *λ*_*opt*_ on the first and second imaging slices, respectively.

For the first and third imaging slices, Fig. 8(a) shows the recovered EEC and Fig. 8(b) shows the diagonal components of the reconstructed conductivity tensors images using *λ*_*opt*_, respectively.

## Discussion

In this paper, we adopt the linear relation between the conductivity tensor and the water diffusion tensor to recover the EEC by combining the diffusion-tensor MRI and MREIT techniques without any referred extracellular information. However, the linear dependence between the diffusion and conductivity tensors has not been well proven to date. To investigate the linear relationship, we recovered the axial anisotropic conductivity, $$\tilde{\sigma }=(\begin{array}{ll}{\sigma }_{11} & {\sigma }_{12}\\ {\sigma }_{12} & {\sigma }_{22}\end{array})$$, from the projected current densities, **J** ^*P,I*^, *i* = 1, 2, by solving the following over-determined matrix equation^33^:

4 $${\bf{Ux}}={\bf{b}}$$

where

$${\bf{U}}=(\begin{array}{lll}\frac{\partial {u}_{1}}{\partial x} & \frac{\partial {u}_{1}}{\partial y} & 0\\ 0 & \frac{\partial {u}_{1}}{\partial x} & \frac{\partial {u}_{1}}{\partial y}\\ \frac{\partial {u}_{2}}{\partial x} & \frac{\partial {u}_{2}}{\partial y} & 0\\ 0 & \frac{\partial {u}_{2}}{\partial x} & \frac{\partial {u}_{2}}{\partial y}\end{array}),\,{\bf{x}}=(\begin{array}{l}{\sigma }_{11}\\ {\sigma }_{12}\\ {\sigma }_{22}\end{array}),\,{\rm{and}}\,{\bf{b}}=(\begin{array}{l}-{J}_{x}^{P\mathrm{,1}}\\ -{J}_{y}^{P\mathrm{,1}}\\ -{J}_{x}^{P\mathrm{,2}}\\ -{J}_{y}^{P\mathrm{,2}}\end{array})\mathrm{.}$$

**Figure 9:**
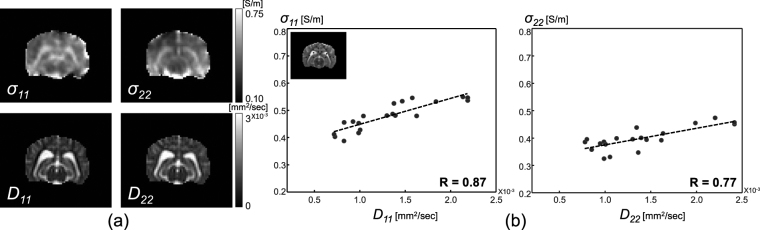
(**a**) Upper row: diagonal components of the reconstructed conductivity tensor by solving matrix system (4), bottom row: diagonal components of the water diffusion tensor. (**b**) Linear relationship between the estimated axial conductivity and water diffusion tensors.

The upper row of Fig. 9(a) shows the diagonal components of the reconstructed conductivity tensors by solving the matrix system (4) without assuming the linear relationship between the conductivity and water diffusion tensors. The bottom row of Fig. 9(a) displays the corresponding water diffusion tensors. Figure 9(b) shows the linear relationship between the estimated axial conductivity and the water diffusion tensors. The correlation coefficient between the diagonal components *σ*_11_ and *D*_11_ was 0.87. For the diagonal components *σ*_22_ and *D*_22_, the correlation coefficient was 0.77.

By applying the MREIT technique and the water molecule apparent diffusion coefficient to therapeutic methods, we can predict the current pathway, electric field distribution, and anisotropic conductivity tensor, which could be useful information for proving the therapeutic effects of electrical stimulation. For EBS using a frequency stimulation of below several kHz, the measured magnetic flux density caused by EBS mainly reflects the ECS effects. Typically, the multi-compartment model in diffusion MRI has been used to explain the transportation phenomenon in human brain tissues. The ADC according to multi *b*-values leads to a two compartment model, such as the following:

$$\frac{S(b)}{S\mathrm{(0)}}={f}_{slow}{e}^{-b\cdot AD{C}_{slow}}+{f}_{fast}{e}^{-b\cdot AD{C}_{fast}}$$

where *S*(*b*) and *S*(0) are the signal with and without the diffusion sensitization gradient, respectively, *ADC*_*slow*_ and *ADC*_*fast*_ represent the fast and slow ADCs, and *f*_*slow*_ and *fast* = 1 − *f* ^*slow*^ are the volume fractions of the fast and slow diffusion, respectively. However, the assignment of the slow and fast diffusion fraction is not straightforward with respect to the intra- and extra-cellular spaces^17,34^. The standard diffusion tensor using a simplified model has some limitations because of its simplicity in explaining complex tissue features^20^. A variety of alternative diffusion models for biological tissues have been developed over the past decade to overcome the limitations of using the diffusivity property^35^. The water diffusion in the extracellular space is less restricted and hindered than that in the intracellular space. The *b*-value is a key parameter in the water diffusion and high *b*-values have been known to be more sensitive to the intracellular space^36^. Although we used the water diffusion tensor with a relatively low *b*-value (800 s/mm^2^), the measured diffusion coefficients still reflect both the intracellular and extracellular spaces because water molecules can move between them. For the anisotropic conductivity tensor combined with the water diffusion process, analyzing the measured diffusion tensor data combined with frequency-dependent electrical properties is a challenging and promising research area.

The noise standard deviation of the measured magnetic flux density satisfies the following relation:

5 $$s{d}_{{B}_{z}}({\bf{r}})=\frac{s{d}_{M}}{2\tilde{\gamma }{T}_{c}M({\bf{r}})}$$

where $\tilde{\gamma }\,=\,26.75\times {10}^{7}$ rad T^−1^ *s* ^−1^ is the gyromagnetic ratio of the proton, *T*_*c*_ is the current injection time width, *M* is the intensity of the MR magnitude image, and *sd*_*M*_ is the noise standard deviation of *M*. The noise level of *B*_*z*_ mainly depends on the time width of the current injection and the field inhomogeneity artifact. In this paper, we used a multi-echo spin-echo MR pulse sequence for the phantom experiments and a multi-echo gradient echo MR pulse sequence for the animal experiment. We can exchange the MR pulse sequences for the experiments based on the use of the multi-echo to prolong the current injection time.

The estimated conductivity values in the ROIs and the ratio of the concentrations across the different ROIs for the two phantom experiments seem to deviate from the directly measured values in Table 1. The EEC and conductivity tensor images in Figs 7 and 8 show some “edge enhancement” effects close to the CSF regions. The measured *B*_*z*_ data are inherently continuous because the current density **J** is related to the measured *B*_*z*_ data by

6 $${B}_{z}({\bf{r}})=\frac{{\mu }_{0}}{4\pi }{\int }_{{\rm{\Omega }}}\frac{(y-y^{\prime} ){J}_{x}({\bf{r}}\text{'})-(x-x^{\prime} ){J}_{y}({\bf{r}}\text{'})}{|{\bf{r}}-{\bf{r}}{\text{'}|}^{3}}d{\bf{r}}\mathrm{\text{'}.}$$

Compared to the ADC, the reconstruction procedure of **J** ^*P*^ requires numerical differentiations of noisy measured *B*_*z*_ data, which may produce some blurring effects. For these reasons, the EEC using the reconstructed **J** and diffusion tensor maps can generate edge enhancement effects in the edge regions. The quality of the reconstructed conductivity is influenced by several complex sources, including the reconstruction procedure for the projected current density from noisy *B*_*z*_ data, the measured diffusion tensor, the approximated relation between the conductivity map and the diffusion tensor map in (15), and the recovery procedure for the ion concentration in (16). Due to the complex procedures and measured noise artifacts, estimating an accurate conductivity map remains a challenge.

**Figure 10:**
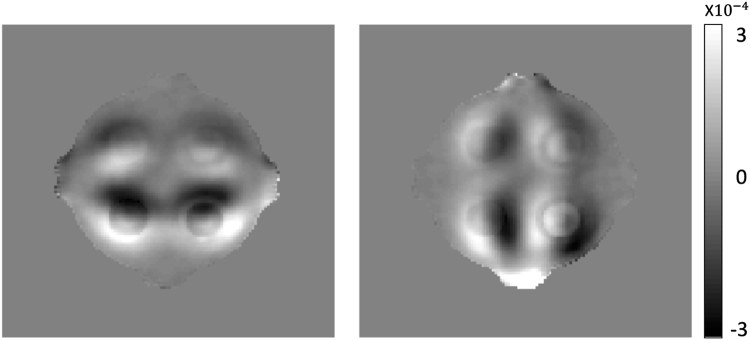
Recovered ${\nabla }^{2}{B}_{z}^{i}$ (*i* = 1, 2) images, which correspond to external current injections of 10 mA for phantom-1, respectively.

For the isotropic conductivity in the phantom experiments, the measured magnetic flux density *B*_*z*_ satisfies the following relationship between the conductivity and electric field:

7 $$(\frac{\partial \sigma }{\partial x},\frac{\partial \sigma }{\partial y})\cdot (\frac{\partial u}{\partial y},-\frac{\partial u}{\partial x})=\frac{1}{{\mu }_{0}}{\nabla }^{2}{{\rm{B}}}_{z}$$

where *u* is the voltage potential. If the conductivity values are homogeneous in each ROI region, relation (7) implies that the harmonic map should be zero in each ROI region because the conductivity values are constant $(\frac{\partial \sigma }{\partial x}=\frac{\partial \sigma }{\partial y}=0)$ in the ROIs. However, the harmonic map ${\nabla }^{2}{B}_{z}$ in Fig. 10 shows a slope change around each ROI region. The contrast of harmonic map means that the conductivity values in Table 1, measured before the phantom experiment using the four electrode method, were changed during the MR scan by the interactions of the ions in each region. This environment change seems to be a main reason for the deviated conductivity values in the ROIs from the directly measured values in Table 3.

For the reconstructed ion concentration images in phantom-2, the profile of the effective ion-concentration shows slight differences in the values in Fig. 4(f) for A and B anomalies despite the same amount of NaCl and choline. Because of the difference in the activity coefficients of choline and NaCl, the effective ion-concentration *η* with respect to the water molecule diffusion coefficient was different at A and B. The anomaly at position C was a mixture of NaCl and choline in a 2:1 ratio. The measured conductivity values of A, B, and C (Fig. 1(b)) were 0.74 (A), 0.63 (B), and 1.63 (C) S/m, respectively. The conductivity value of C was lower than the expected value. Together with the specific requirements of moving ions, such as viscosity and electrophoretic effects, Debye and Huckel showed the conductivity values in dilute solution relate to the mean activity coefficient as a function of the ionic strength^37^. The measured magnetic flux density by the low-frequency EBS reflects the extracellular electrical current density. The proposed method can estimate only the apparent total electrolyte concentrations compared to the water molecule mobility term because DWI measures the random movement of the water molecules in tissues. The proposed EEC imaging in the brain region requires a more rigorous analysis that combines the activity coefficient and the extracellular diffusion coefficient.

In this paper, we required two independent injection currents to recover the EEC because the proposed reconstruction algorithm is based on the matrix system (17). However, conventional EBSs typically have a single injection current source. To be a more practical clinical device, it would be desirable to develop a method for EEC that uses only one injection current.

## Conclusion

In this paper, we propose a novel technique to visualize the total electrolyte concentration in the extracellular compartment of the brain on an imaging slice. Since the electrical conductivity can be decomposed into the concentrations and mobilities of the ions, combining with the diffusion tensor MRI (DT-MRI) and MREIT techniques, we designed a fast non-iterative technique to visualize the total extracellular electrolyte concentration (EEC). Through two separate phantom experiments in which we controlled the concentrations and mobilities of the ions, we verified that the proposed method can extract the ion-concentration information using conventional MR imaging pulse sequences. We also performed *in vivo* canine brain EEC imaging. Due to the EEC distribution in the brain region, the electrical characteristics, including the current pathway, electric field distribution, apparent ions velocities in the electric field, and anisotropic conductivity tensor were investigated to demonstrate the therapeutic effects of EBS.

## Theory

### Effective ion concentration in the extracellular space

The effective electrical conductivity tensor of the two-phase anisotropic medium (intra- and extra-cellular spaces) shares the same eigenvectors as the water diffusion tensor in the brain tissue^21^. The equivalence between the conductivity and diffusion tensor eigenvectors makes it possible to express cross-property relationships in terms of the conductivity and diffusion tensor eigenvalues. Thin insulating cell membranes block low-frequency currents (<1 kHz), so the intracellular space is electrically shielded by cell membranes. For low-frequency conductivity, the eigenvalue ${c}_{{\lambda }_{i}}$ of the conductivity tensor C_*σ*_ perturbs the eigenvalue ${{d}}_{{\lambda }_{i}}$ of the water diffusion tensor D and can be expressed as

$${c}_{{\lambda }_{i}}=\frac{{\sigma }_{e}}{{d}_{e}}[{d}_{{\lambda }_{i}}(\frac{{d}_{i}}{3{d}_{e}}+1)+\frac{{d}_{{\lambda }_{i}}^{2}{d}_{i}}{3{d}_{e}^{2}}-\frac{2}{3}{d}_{i}]+{\mathscr{O}}({d}_{i}^{2}),\,i=\mathrm{1,}\,\mathrm{2,}\,3$$

where *σ*_*e*_ is the extracellular conductivity, *d*_*i*_ and *d*_*e*_ are the intra- and extra-cellular diffusion coefficients, respectively, and ${\mathscr{O}}({d}_{i}^{2})$ is bounded as ${d}_{i}^{2}$ tends to infinity^23^.

Depending on the ion types (i.e. the activity coefficient *γ*), concentration (*c*), and mobility (*μ*), the electrical conductivity (*σ*) of an electrolyte solution can be expressed as

8 $$\sigma ={A}_{\nu }qz\gamma c\mu $$

where *A*_*v*_ = 6.02 × 10^23^ l/mol is Avogardro’s number, *q* = 1.6 × 10^−19^ C is the absolute value of the charge of a single electron, and *z* is the charge number of ion. The two compartment model (the intra- and extra-cellular mediums) is typically used in the study of the transportation process for biological tissues where the cells are submerged in the extracellular fluid and separated by a thin insulating membrane. It should be noted that both the intra- and extra-cellular spaces contain the electrolyte solution so the equation (8) is valid for both compartments. At a low frequency probing current (a frequency of below 1 kHz), the electrical conductivity is only determined by the conductivity at the extracellular space because the membrane at that frequency acts as an insulator.

The low frequency conductivity tensor map in a voxel can be expressed as a sum of products of the carrier concentration and the mobility tensor. The low-frequency conductivity in biological tissues is

9 $${{\bf{C}}}_{\sigma }=\sum _{j=1}^{M}{A}_{\nu }q{z}^{j}{\gamma }_{e}^{j}{c}_{e}^{j}{{\bf{M}}}_{e}^{j}$$

where *z* ^*j*^ is the charge number, ${\gamma }_{e}^{j}$ is the extracellular activity coefficient, ${c}_{e}^{j}$ is the extracellular concentration, and ${{\bf{M}}}_{e}^{j}$ is the extracellular mobility tensor of the *j* th charge carrier. For a reference charge carrier (for example ${{\bf{M}}}_{e}^{1}$), we express the low-frequency conductivity (9) as

$${{\bf{C}}}_{\sigma }=\sum _{j=1}^{M}{A}_{\nu }q{z}^{j}{\gamma }_{e}^{j}{c}_{e}^{j}{k}^{j}{{\bf{M}}}_{e}^{1}$$

where $${{\bf{M}}}_{e}^{j}={k}^{j}{{\bf{M}}}_{e}^{1}$$ and *k* ^1^ = 1.

The effective water diffusion tensor **D** ^*w*^ can be written as a positive definite symmetric matrix:

10 $${{\bf{D}}}^{w}={S}_{D}{{\bf{D}}}_{\lambda }^{w}{{\bf{S}}}_{D}^{T}\,\,{\rm{with}}\,{{\bf{D}}}_{\lambda }^{w}=(\begin{array}{lll}{d}_{{\lambda }_{1}}^{w} & 0 & 0\\ 0 & {d}_{{\lambda }_{2}}^{w} & 0\\ 0 & 0 & {d}_{{\lambda }_{3}}^{w}\end{array})$$

where the column vectors of **S** ^*D*^ are the orthogonal eigenvectors of **D** ^*w*^, the superscript *T* denotes the transpose and ${d}_{{\lambda }_{i}}^{w}\mathrm{,\ }i=\mathrm{1,}\,\mathrm{2,}\,\mathrm{3,}$ are the corresponding eigenvalues.

The effective low-frequency conductivity tensor of a macroscopic voxel for the reference charge carrier is given by

11 $${{\bf{C}}}_{\sigma }={{\bf{S}}}_{D}{{\bf{C}}}_{\lambda }{{\bf{S}}}_{D}^{T}=\sum _{j=1}^{M}{A}_{\nu }q{z}^{j}{\gamma }_{e}^{j}{c}_{e}^{j}{k}^{j}{{\bf{M}}}_{e}^{1}\,\,{\rm{with}}\,{{\bf{C}}}_{\lambda }=(\begin{array}{lll}{c}_{{\lambda }_{1}} & 0 & 0\\ 0 & {c}_{{\lambda }_{2}} & 0\\ 0 & 0 & {c}_{{\lambda }_{3}}\end{array})\mathrm{.}$$

From Einstein’s relationship, in which the diffusion coefficient of moving particles is related to the mobility, the water diffusion tensor can be linked to the reference charge transport property:

12 $${{\bf{M}}}_{e}^{1}=\frac{q}{{k}_{B}{T}_{a}}{{\bf{D}}}_{e}^{1}=\frac{Kq}{{k}_{B}{T}_{a}}{{\bf{D}}}^{w}$$

where *k*_*B*_ is Boltzman’s constant, *T* ^*a*^ is the absolute temperature, and *K* is a constant between ${{\bf{D}}}_{e}^{1}$ and the water diffusion tensor **D** ^*w*^.

Combining (10), (11), and (12), we have

13 $${{\bf{C}}}_{\sigma }={{\bf{S}}}_{D}{{\bf{C}}}_{\lambda }{{\bf{S}}}_{D}^{T}=\eta {{\bf{S}}}_{D}{{\bf{D}}}_{\lambda }^{w}{{\bf{S}}}_{D}^{T},\,\eta \,:=\frac{Kq}{{k}_{B}{T}_{a}}\sum _{j\mathrm{=1}}^{M}{A}_{\nu }q{z}^{j}{\gamma }_{e}^{j}{c}_{e}^{j}{k}^{j}(:=\sum _{j\mathrm{=1}}^{M}{\delta }^{j}{c}_{e}^{j})\mathrm{.}$$

In relation (13), we call *η* the total effective electrolyte concentration (EEC) and *Kk* ^*j*^ is the normalization constant for the *j*-th ionic species (for the reference charge carrier, *k* ^1^ = 1). Here, we use the term “effective” because of the normalization constants *Kk* ^*j*^ and the activity coefficient ${\gamma }_{e}^{j}$. According to the Debye-Huckel theory for the electrolyte solution, the activity coefficient depends on the ionic strength and hydration radius (Stokes radius). For a given ionic strength, the activity coefficient only depends on the hydration radius, which is further related with the size of ions. Debye-Huckel theory predicts that the activity coefficient increases as the hydration radius increases and vice versa.

The representative diffusion coefficient is not identical to the extracellular diffusion coefficient, but a diffusion tensor coefficient at a relatively low *b*-value (<1000 s/mm^2^) dominates the fast diffusion component in each direction. Based on the fact that fast diffusion is proportional to the extracellular diffusion, we approximate the diffusion tensor at low *b*-values by the extracellular diffusion tensor:

$${{\bf{D}}}^{w}=\alpha {{\bf{D}}}_{e}^{w}+\mathrm{(1}-\alpha ){{\bf{D}}}_{i}^{w}\approx \alpha {{\bf{D}}}_{e}^{w}\mathrm{.}$$

For the anisotropic conductivity tensor **C**_*σ*_, the divergence-free condition of current density and applied external current density satisfy

$$\{\begin{array}{llll}\nabla \cdot {\bf{J}}=-\nabla \cdot ({{\bf{C}}}_{\sigma }\nabla u) & = & 0 & \,{\rm{in}}\,{\rm{\Omega }}\\ {\bf{J}}\cdot \nu =-{{\bf{C}}}_{\sigma }\nabla u\cdot \nu  & = & g & {\rm{on}}\,\partial {\rm{\Omega }}\mathrm{.}\end{array}$$

The relation (13) implies that

$$\nabla \cdot ({{\bf{C}}}_{\sigma }\nabla u)=\nabla \cdot ((\eta {S}_{D}{\bf{D}}{{\bf{S}}}_{D}^{T})\nabla u)=0\,{\rm{in}}\,{\rm{\Omega }}\mathrm{.}$$

### Computation of projected current density

Let Ω be a cylindrical domain with boundary $\partial {\rm{\Omega }}$. We may express Ω as a union of slices perpendicular to the *z*-axis:

$${\rm{\Omega }}={\cup }_{t\in (-H,H)}{{\rm{\Omega }}}_{t}\,{\rm{where}}\,{{\rm{\Omega }}}_{t}={\rm{\Omega }}\cap \{(x,y,z)\in {{\mathbb{R}}}^{3}\mathrm{|\ }z=t\in (-\,H,H)\}\mathrm{.}$$

Here, Ω_0_ denotes the center slice. In this paper, we use the following two-dimensional estimations:

$$\tilde{\nabla }f\,:=(\frac{\partial f}{\partial x},\frac{\partial f}{\partial y},\,0),\,{\tilde{\nabla }}^{\perp }f\,:=(\frac{\partial f}{\partial y},-\frac{\partial f}{\partial x},\,0)\mathrm{.}$$

Since *B*_*z*_ is the only measurable quantity without rotating the object inside the MRI scanner, we inject current in the orthogonal direction to the main magnetic field through a pair of attached electrodes to maximize *B*_*z*_. The projected current density **J** ^*P*^ is the best approximation of **J**, and can be estimated from the measured *B*_*z*_ of **B** induced by the injected current. The projected current density can be expressed in decomposed form:

14 $${{\bf{J}}}^{P}={{\bf{J}}}^{0}+{\tilde{\nabla }}^{\perp }\psi $$

where **J** ^0^ is the current density corresponding to the background conductivity. The potential function *ψ* satisfies

$$\{\begin{array}{llll}{\tilde{\nabla }}^{2}\psi  & = & \frac{1}{{\mu }_{0}}{\nabla }^{2}({B}_{z}-{B}_{z}^{0}) & {\rm{in}}\,{{\rm{\Omega }}}_{t}\\ \psi  & = & 0 & {\rm{on}}\,\partial {{\rm{\Omega }}}_{t}\end{array}$$

where ${{\bf{B}}}^{0}=({B}_{x}^{0},{B}_{y}^{0},{B}_{z}^{0}))$ is the magnetic field corresponding to **J** ^0^.

### Reconstruction algorithm for effective ion concentration

From the approximated relation between the conductivity tensor map **C**_*σ*_ and the diffusion tensor map **D**, the conductivity tensor can be represented as

15 $${{\bf{C}}}_{\sigma }=\eta {\bf{D}}$$

where the scale factor *η* reflects EEC information^22^. From the measured ${B}_{z}^{i}$ data corresponding to external injection currents, ${I}_{i}^{\pm }\mathrm{,\ }\,i=\mathrm{1,}\,2$, the recovered internal currents **J** ^*i*^ can be expressed by

$${{\bf{J}}}^{i}=-{{\bf{C}}}_{\sigma }\nabla {u}^{i}=-\eta {\bf{D}}\nabla {u}^{i}$$

where *u* ^*i*^ is the voltage potential and *η* is a scaling factor to be determined from the apparent current density **J** ^*i*^, *i* = 1, 2.

The diffusion tensor map **D**, the current **J** ^*i*^ and the scale factor *η* directly satisfy the following relation

16 $$\nabla \times ({{\bf{D}}}^{-1}{{\bf{J}}}^{i})=-\frac{\nabla \eta }{\eta }\times (\eta \nabla {u}^{i})=\nabla \,\mathrm{log}\,\eta \times ({{\bf{D}}}^{-1}{{\bf{J}}}^{i})$$

Relation (16) leads to the following matrix system

17 $$(\begin{array}{cc}{E}_{y}^{1} & -{E}_{x}^{1}\\ {E}_{y}^{2} & -{E}_{x}^{2}\end{array})(\begin{array}{c}\frac{\partial \,\mathrm{ln}\,\eta }{\partial x}\\ \frac{\partial \,\mathrm{ln}\,\eta }{\partial y}\end{array})=(\begin{array}{c}\frac{\partial {E}_{y}^{1}}{\partial x}-\frac{\partial {E}_{x}^{1}}{\partial y}\\ \frac{\partial {E}_{y}^{2}}{\partial x}-\frac{\partial {E}_{x}^{2}}{\partial y}\end{array})$$

where ${E}_{x}^{i}$ and ${E}_{y}^{i}$ are the *x*- and *y*-components of ${{\bf{D}}}^{-1}{{\bf{J}}}^{i}$, respectively. Each component of the above matrices includes non-negligible noise artifacts due to a small amount of injection current and the inversion process of the diffusion tensor **D**. We first introduce a search neighborhood ${{\mathscr{N}}}_{(x,y)}=\{({x}_{i},{y}_{i}):i=\mathrm{1,}\cdots ,N\}$ around (*x*, *y*) and set a weighting factor as

$${w}_{i}={e}^{-h||S({x}_{i},{y}_{i})-S(x,y)||}/\sum _{j=1}^{N}{e}^{-h||S({x}_{j},{y}_{j})-S(x,y)||}$$

where *S* is the magnitude of the MR image and *h* is a function of the noise level of *B*_*z*_.

Next, let’s consider the regularized weighted least squares problem

18 $$\mathop{{\rm{\min }}}\limits_{{\bf{x}}(x,y)}\frac{1}{2}||{\bf{A}}(x,y)x(x,y)-{\bf{b}}(x,y)|{|}^{2}+\frac{\lambda }{2}||{\bf{x}}(x,y)|{|}^{2}$$

where *λ* is the regularization parameter,

$${\bf{A}}=(\begin{array}{cc}{w}_{1}{E}_{y}^{1}({x}_{1},{y}_{1}) & -{w}_{1}{E}_{x}^{1}({x}_{1},{y}_{1})\\ {w}_{1}{E}_{y}^{2}({x}_{1},{y}_{1}) & -{w}_{1}{E}_{x}^{2}({x}_{1},{y}_{1})\\ \vdots  & \vdots \\ {w}_{N}{E}_{y}^{1}({x}_{N},{y}_{N}) & -{w}_{N}{E}_{x}^{1}({x}_{N},{y}_{N})\\ {w}_{N}{E}_{y}^{2}({x}_{N},{y}_{N}) & -{w}_{N}{E}_{x}^{2}({x}_{N},{y}_{N})\end{array}),{\bf{b}}=(\begin{array}{l}{w}_{1}(\tfrac{\partial {E}_{y}^{1}({x}_{1},{y}_{1})}{\partial x}-\tfrac{\partial {E}_{x}^{1}({x}_{1},{y}_{1})}{\partial y})\\ {w}_{1}(\tfrac{\partial {E}_{y}^{2}({x}_{1},{y}_{1})}{\partial x}-\tfrac{\partial {E}_{x}^{2}({x}_{1},{y}_{1})}{\partial y})\\ \,\,\,\,\,\,\,\,\,\,\,\,\,\,\,\,\,\,\,\,\,\,\,\,\vdots \\ {w}_{N}(\tfrac{\partial {E}_{y}^{1}({x}_{N},{y}_{N})}{\partial x}-\tfrac{\partial {E}_{x}^{1}({x}_{N},{y}_{N})}{\partial y})\\ {w}_{N}(\tfrac{\partial {E}_{y}^{2}({x}_{N},{y}_{N})}{\partial x}-\tfrac{\partial {E}_{x}^{2}({x}_{N},{y}_{N})}{\partial y})\end{array})\,{\rm{and}}\,{\bf{x}}={(\tfrac{\partial ln\eta }{\partial x}\tfrac{\partial ln\eta }{\partial y})}^{T}.$$

To solve the weighted least square problem (18), it is crucial to determine an optimal regularization parameter *λ* for a successful EEC and conductivity reconstruction. To determine the regularization parameter *λ*, we use the GCV technique, which minimizes the predictive mean-square error without statistical information^38^.

Let $A=U{\rm{\Sigma }}{V}^{T}$ be the singular value decomposition of the 2*N* × 2 matrix **A** with singular values *s*_1_, *s*_2_. The GCV function is

19 $$GCV(\lambda \mathrm{)=2}\sum _{i\mathrm{=1}}^{2}{(\frac{{\hat{b}}_{i}}{{s}_{i}^{2}+\lambda })}^{2}/{(\sum _{i\mathrm{=1}}^{2}\frac{1}{{s}_{i}^{2}+\lambda })}^{2}$$

where $\hat{b}$ = *U* ^*T*^ **b**. Since GCV(*λ*) is a continuous function, we can find the optimal *λ*_*opt*_ at which GCV is minimized. With this optimal parameter *λ*_*opt*_, we use the singular value decomposition of *A* to find the solution of the regularized least squares problem (18).
